# Final analysis of the phase 3 randomized clinical trial comparing HD201 vs. referent trastuzumab in patients with ERBB2-positive breast cancer treated in the neoadjuvant setting

**DOI:** 10.1186/s12885-023-10574-2

**Published:** 2023-01-31

**Authors:** Xavier Pivot, Alexey Georgievitch Manikhas, Volodymyr Shamrai, Giorgi Dzagnidze, Hwoei Fen Soo Hoo, Viriya Kaewkangsadan, Fausto Petrelli, Cristian Villanueva, Jamie Kim, Sumita Pradhan, Litha Jaison, Peggy Feyaerts, Leonard Kaufman, Marie-Paule Derde, Filip Deforce, David G. Cox

**Affiliations:** 1 Institute of Cancer Strasbourg, 17 Rue Albert Calmette, 67033 Strasbourg, France; 2 St Petersburg GBUZ City Clinical Oncology Dispensary, St Petersburg, Russia; 3 Vinnytsia Regional Clinical Oncological Dispensary, Vinnytsia, Ukraine; 4 S. Khechinashvili University Hospital, Tbilisi, Georgia; 5 Penang General Hospital, Penang Island, Malaysia; 6 Department of Surgery, Phramongkutklao Hospital, Bangkok, Thailand; 7 Oncology Unit, ASST Bergamo Ovest, Bergamo, Trevigilio Italy; 8 Clinique Clementville, Montpellier, Montpellier, France; 9 Prestige BioPharma Ltd, Singapore, Singapore; 10 DICE, Naamloze Vennootschap, Dilbeek, Belgium

**Keywords:** Trastuzumab, Biosimilar, HD201, Breast cancer, Neoadjuvant, HER2, ERBB2

## Abstract

**Background:**

The TROIKA trial established that HD201 and trastuzumab were equivalent in terms of primary endpoints (total pathological complete response) following neoadjuvant treatment. The objective of the present analysis was to compare survival outcomes and final safety.

**Methods:**

In the TROIKA trial, patients with ERBB2-positive early breast cancer were randomized and treated with either HD201 or the referent trastuzumab. Eligible patients received 8 cycles of either HD201 or referent trastuzumab (loading dose, 8 mg/kg; maintenance dose, 6 mg/kg) every 3 weeks in combination with 8 cycles of chemotherapy (4 cycles of docetaxel, 75 mg/m^2^, followed by 4 cycles of epirubicin, 75 mg/m^2^, and cyclophosphamide, 500 mg/m^2^) in the neoadjuvant setting. The patients then underwent surgery followed by 10 cycles of adjuvant HD201 or referent trastuzumab according to their initial randomization to complete one year of trastuzumab-directed therapy. Event-free and overall survival rates were calculated using Kaplan–Meier analysis. The hazard ratio for event-free survival was estimated by Cox proportional hazards regression.

**Results:**

The final analysis was performed after all patients completed the study at a median follow-up of 37.7 months (Q1-Q3, 37.3–38.1 months). A total of 502 randomized patients received either HD201 or the referent trastuzumab, and 474 (94.2%) were eligible for inclusion in the per-protocol set. In this population, the 3-year event-free survival rates were 85.6% (95% CI: 80.28–89.52) and 84.9% (95% CI: 79.54–88.88) in the HD201 and referent trastuzumab groups, respectively (log rank *p* = 0.938) (HR 1.02, 95% CI: 0.63–1.63; *p* = 0.945). The 3-year overall survival rates were comparable between the HD201 (95.6%; 95% CI: 91.90–97.59) and referent trastuzumab treatment groups (96.0%, 95% CI: 92.45–97.90) (log rank *p* = 0.606). During the posttreatment follow-up period, adverse events were reported for 64 (27.4%) and 72 (29.8%) patients in the HD201 and the reference trastuzumab groups, respectively. Serious adverse events were rare and none of which were related to the study treatment.

**Conclusions:**

This final analysis of the TROIKA trial further confirms the comparable efficacy and safety of HD201 and trastuzumab.

**Trial registration:**

ClinicalTrials.gov identifier: NCT03013504.

## Introduction

In the primary analysis of the prospective, randomized, multicenter phase 3 TROIKA study, HD201, a trastuzumab biosimilar, was shown to be equivalent to the referent trastuzumab in patients with ERBB2-positive early breast cancer (EBC) based on the primary endpoints of locally assessed total pathologic complete response (tpCR) [1].

The relationship between tpCR status and survival has been extensively debated following a meta-analysis indicating that tpCR status predicts survival outcome in patients with ERBB2-positive EBC [2]. Regulatory agencies have acknowledged this relationship by authorizing several compounds on this early criterion for activity [3–7]. The neoadjuvant setting can be definitively considered the new era for development in ERBB2-positive breast cancer [8]. It remains reassuring that in most cases, the conclusion derived from the early criteria of pathologic complete response (pCR) has been confirmed by survival outcome analysis [9–11]. In this final analysis of the TROIKA study, we report the long-term efficacy and safety outcomes at 3 years of follow-up.

## Methods

### Study design and patients

TROIKA (NCT03013504) was a multicenter, randomized, phase 3 trial previously detailed in the publication reporting the primary analysis [1]. The study was performed in accordance with the Declaration of Helsinki and Good Clinical Practice guidelines. Approval of the study protocol and all accompanying documents provided to the patients was obtained from independent ethics committees at participating institutions, and all patients provided voluntary written informed consent. Key eligibility criteria were age ≥ 18 years; ERBB2-positivity; new diagnosis; unilateral, operable breast cancer; and a baseline left ventricular ejection fraction ≥ 55%.

Patients were enrolled and randomized using a block of 8 in a ratio of 1:1 to receive either HD201 or referent trastuzumab (loading dose: 8 mg/kg; maintenance dose: 6 mg/kg) every 3 weeks, administered concurrently with 8 cycles of chemotherapy (4 cycles of docetaxel [75 mg/m^2^], followed by 4 cycles of epirubicin [75 mg/m^2^]/cyclophosphamide [500 mg/m^2^]) in the neoadjuvant setting. After surgery, patients received an additional 10 cycles of HD201 or referent trastuzumab in the adjuvant setting according to the previous allocation.

### Outcomes

Secondary objectives included evaluation of event-free survival (EFS) (defined as the time from randomization to the first observation of disease progression, including local and distant recurrence, second primary cancer, or death due to any cause), overall survival (OS) (defined as the time from randomization to death), safety, and immunogenicity. Exploratory analyses were conducted for EFS including locally assessed tpCR and bpCR as covariates.

### Statistical analysis

**Fig. 1:**
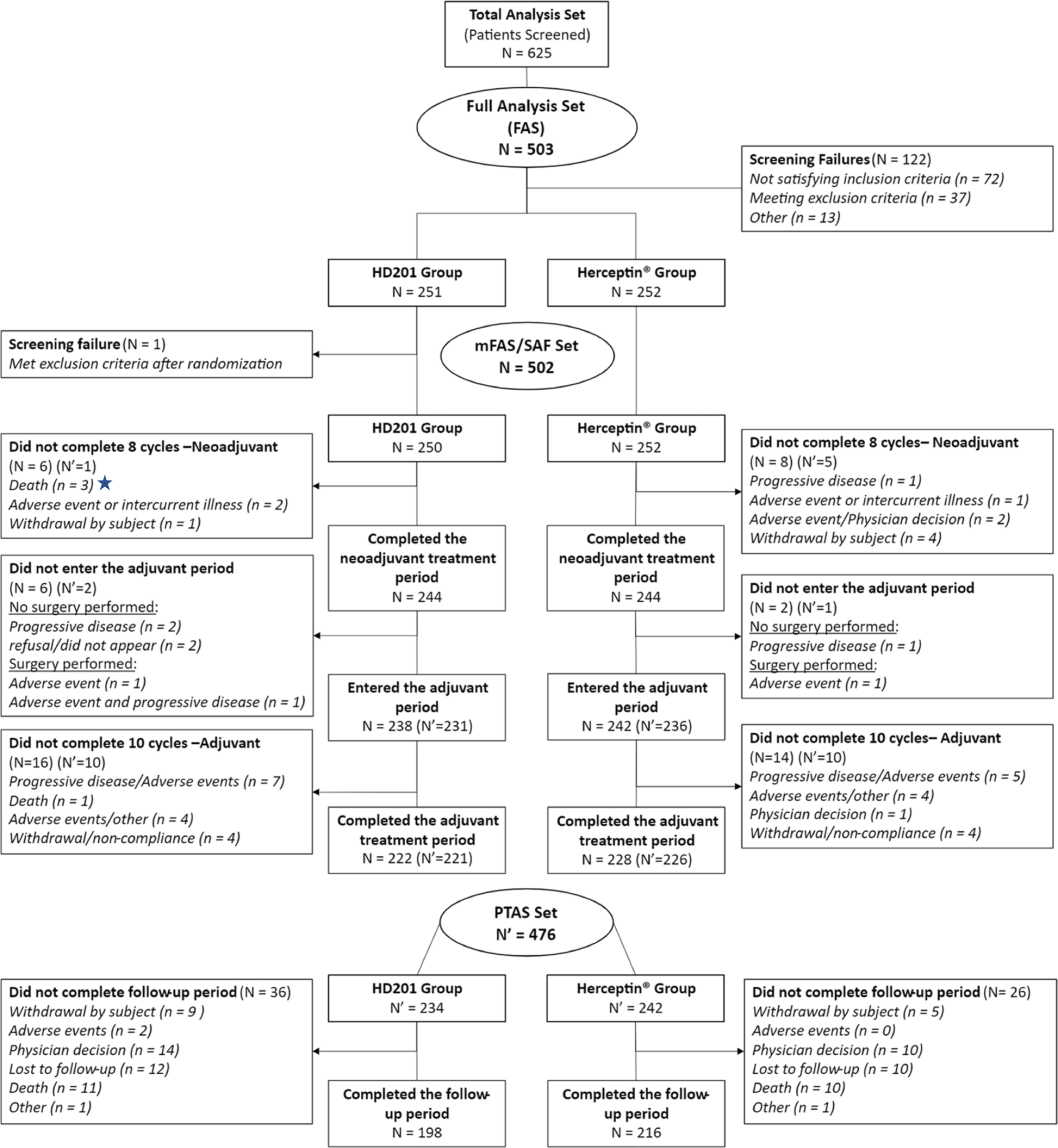
Patient distribution: CONSORT diagram

Target sample sizes and statistical power calculations for the primary analysis have been reported previously [1]. Statistical analyses were performed with SAS (version 9.4; SAS Institute Inc., NC, USA). Kaplan–Meier analysis was used to estimate EFS and OS rates. Cox proportional hazards regression analyses providing hazard ratios (HRs) and 95% confidence intervals (95% CIs) for EFS adjusted for region, stage, and tumor hormonal receptor status are presented. Survival analyses were conducted in the per-protocol set (PPS), including all patients who received the study treatment (without a major protocol deviation affecting the primary efficacy assessment) and who underwent surgery after the completion of neoadjuvant treatment or did not undergo surgery because of lack of efficacy, and analysis was also performed in the modified full analysis set (mFAS), including all patients who received at least 1 dose of study medication (Fig. 1). Safety analyses were descriptive and conducted in all patients who received at least one dose of treatment. Adverse events (AEs) and serious AEs (SAEs) were recorded and graded per standard common technology criteria for adverse events (CTCAE).

## Results

### Patient population

This analysis was performed after all patients completed the study at a median follow-up of 37.7 months (Q1-Q3, 37.3–38.1 months). The mFAS comprised 502 randomized and treated patients, among whom 250 (49.8%) were in the HD201 group and 252 (50.2%) were in the referent trastuzumab group and were included between February 19 and September 21, 2018, across 70 centers in 12 countries. A total of 28 patients with mFAS were excluded from the PPS (12 patients in the HD201 treatment group and 16 patients in the referent trastuzumab group). The PPS thus comprised 238 patients in the HD201 treatment group and 236 patients in the referent trastuzumab treatment group. Baseline demographics and disease characteristics were well balanced between the study arms as reported previously [1].

### Efficacy

**Fig. 2:**
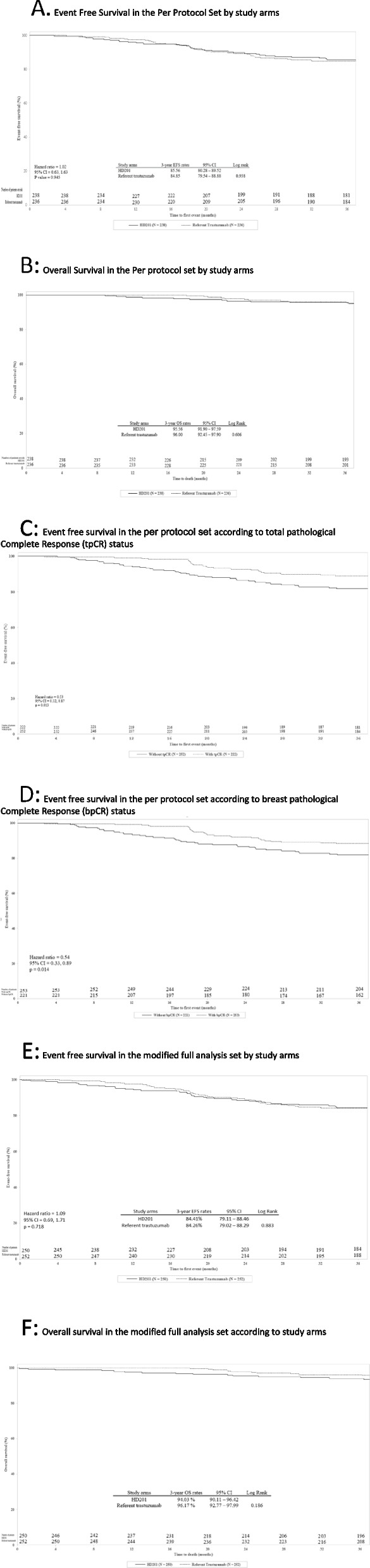
Event-Free Survival and Overall Survival in the Per Protocol set (PPS) and in the modified Full Analysis set (mFAS). **A** EFS by study arm in the PPS. **B** OS by study arm in the PPS. **C** EFS by tpCR status in the PPS. **D** EFS by bpCR status in the PPS. **E** Event free survival in the mFAS. **F** Overall survival in the mFAS. bpCR, breast pathologic complete response; CI, confidence interval; EFS, event-free survival; HR, hazard ratio; PPS, per protocol set; OS, overall survival; pCR, pathologic complete response; tpCR, total pathologic complete response

In the PPS, the 3-year EFS rates were 85.6% (95% CI: 80.28–89.52) and 84.9% (95% CI: 79.54–88.88) in the HD201 and referent trastuzumab groups, respectively (log rank *p* = 0.938) (Fig. 2A). The Cox proportional HR adjusted for region, stage, and tumor hormonal receptor status was 1.02 (95% CI: 0.63–1.63; *p* = 0.945) (Fig. 2A). The 3-year OS rates were comparable for the HD201 (95.5%; 95% CI: 91.90–97.59) and referent trastuzumab treatment groups (96.0%, 95% CI: 92.45–97.90) (log rank *p* = 0.606) (Fig. 2B). These results for EFS and OS were similar to those in the mFAS population (Figs. 2E and F). The sensitivity analysis searching heterogeneity of treatment effect according to the disease characteristics did not observed any discordances between the two arms in terms of survival outcomes.

### Locally assessed pCR and long-term efficacy

In the PPS, in both treatment arms, 3-year EFS was more better for patients achieving a tpCR (locally assessed) than for those with residual disease, with 10.8% (24/222) versus 17.9% (45/252) of patients with events counting for EFS, respectively (HR 0.53, 95% CI 0.32–0.87; *p* = 0.013) (Fig. 2C). Similarly, 3-year EFS was more favorable for patients achieving a bpCR (locally assessed) than for those without (HR 0.54, 95% CI 0.33–0.89; *p* = 0.014) (Fig. 2D).

### Long-term safety

During the posttreatment follow-up period, PTAEs were reported for 64 (27.4%) and 72 (29.8%) patients in the HD201 and the referent trastuzumab groups, respectively (Table 1). PTAEs with severity grade 3 or higher were reported for 7 (3.0%) patients and 13 (5.4%) patients, and serious PTAEs were reported for 4 (1.7%) patients and 5 (2.1%) patients, respectively. No serious PTAEs related to study treatment were reported during the posttreatment follow-up period. Overall, no noteworthy differences were found between the two groups.

**Table 1:** Safety results for the post treatment period

	**HD201**	**Herceptin®**
	***N* = 234**	***N* = 242**
**Patients presenting with ANY**	*n* (%)	*n* (%)
PTAE	64 (27.4%)	72 (29.8%)
PTAE Related to Study Treatment	21 (9.0%)	23 (9.5%)
PTAE ≥ Grade 3	7 (3.0%)	13 (5.4%)
Serious PTAE	4 (1.7%)	5 (2.1%)
Serious PTAE Related to Study Treatment	0 (0.0)	0 (0.0)
PTAE of Special Interest	35 (15.0%)	40 (16.5%)
PTAE by Preferred Term		
Cardiac disorders	19 (8.1%)	27 (11.2%)
Neoplasms benign, malignant, and unspecified (incl. cysts and polyps)	16 (6.8%)	14 (5.8%)
Blood and lymphatic system disorders	12 (5.1%)	10 (4.1%)
PTAEs related to study treatment by preferred term		
Cardiac disorders	12 (5.1%)	11 (4.5%)
Blood and lymphatic system disorders	6 (2.6%)	1 (0.4%)

*PTAE* Post treatment Adverse Event

## Discussion

The phase 3 TROIKA study in patients with ERBB2-positive EBC is the conclusive step in the investigation of HD201 and the referent trastuzumab in the extensive comparison of the two supporting the development of the biosimilar candidate [1]. Analysis of the secondary long-term efficacy endpoints, EFS and OS, after 3 years of follow-up continues to support the equivalence of HD201 to referent trastuzumab established by the primary analysis based on the tpCR criterion. Most recurrent events in ERBB2-positive breast cancer have been reported to occur within 3 years, and this duration appears sufficient to provide adequate evidence to support efficacy and safety conclusions [12–14]. Achieving tpCR was associated with longer EFS in both treatment arms, and these results were consistent with those observed in other studies assessing neoadjuvant trastuzumab [9–11, 14].

The overall safety profile of HD201 and trastuzumab at the 3-year follow-up remains consistent with the safety profiles observed in previous studies, post-treatment adverse events are unrelated or unlikely to the study drug, and rarely, events related to the study drug occurred in the post-treatment follow-up period.

Limitations of the study include the use of newer anti-HER2 agents, which could impact survival in patients with relapse and were not assessed in this study. In addition, subgroup analyses are limited by their small and unbalanced sample sizes.

## Conclusions

This final analysis of TROIKA further supports the comparability of the efficacy and safety of HD201 and the referent trastuzumab.

## Data Availability

Data types: Deidentified participant data. How to access data: The application providing the project details should be submitted to Prestige Bio Pharma,(2 Science Park Dr, #04–13/14 Ascent Tower B, Singapore Science Park, Singapore 118,222) or by email at jamie.kim@prestigebio.com or x.pivot@icans.eu. Then the request need to be approved by the steering committee of the study before release the data. Restriction: The steering committee of the trial approval based on the scientific assessment of the application is requested to release the data.
